# Increased circulating interleukin-8 in patients with resistance to thyroid hormone receptor α

**DOI:** 10.1530/EC-17-0213

**Published:** 2017-09-28

**Authors:** Anne H van der Spek, Olga V Surovtseva, Saskia Aan, Anton T J Tool, Annemarie van de Geer, Korcan Demir, Anja L M van Gucht, A S Paul van Trotsenburg, Timo K van den Berg, Eric Fliers, Anita Boelen

**Affiliations:** 1 Department of Endocrinology and MetabolismAcademic Medical Center, Amsterdam, The Netherlands; 2 Sanquin Research and Landsteiner LaboratoryAcademic Medical Center, Amsterdam, The Netherlands; 3 Division of Pediatric EndocrinologyDokuz Eylül University, Izmir, Turkey; 4 Department of EndocrinologyErasmus Medical Center, Rotterdam, The Netherlands; 5 Department of Pediatric EndocrinologyAcademic Medical Center, Amsterdam, The Netherlands

**Keywords:** thyroid hormone receptor alpha, resistance to thyroid hormone, interleukin-8, macrophage, neutrophil, innate immunity

## Abstract

Innate immune cells have recently been identified as novel thyroid hormone (TH) target cells in which intracellular TH levels appear to play an important functional role. The possible involvement of TH receptor alpha (TRα), which is the predominant TR in these cells, has not been studied to date. Studies in TRα^0/0^ mice suggest a role for this receptor in innate immune function. The aim of this study was to determine whether TRα affects the human innate immune response. We assessed circulating interleukin-8 concentrations in a cohort of 8 patients with resistance to TH due to a mutation of TRα (RTHα) and compared these results to healthy controls. In addition, we measured neutrophil and macrophage function in one of these RTHα patients (mutation D211G). Circulating interleukin-8 levels were elevated in 7 out of 8 RTHα patients compared to controls. These patients harbor different mutations, suggesting that this is a general feature of the syndrome of RTHα. Neutrophil spontaneous apoptosis, bacterial killing, NAPDH oxidase activity and chemotaxis were unaltered in cells derived from the RTHαD211G patient. RTHα macrophage phagocytosis and cytokine induction after LPS treatment were similar to results from control cells. The D211G mutation did not result in clinically relevant impairment of neutrophil or pro-inflammatory macrophage function. As elevated circulating IL-8 is also observed in hyperthyroidism, this observation could be due to the high-normal to high levels of circulating T_3_ found in patients with RTHα.

## Introduction

Thyroid hormone (TH) is essential for normal growth and development and largely exerts its biological actions through binding to nuclear thyroid hormone receptors (1). Thyroid hormone receptors (TRs) are encoded by the thyroid hormone receptor α and thyroid hormone receptor β genes (*THRA* and *THRB*, respectively), which can be alternatively spliced into several isoforms that are differentially expressed in various tissue and cell types (2). The two main isoforms of TRα are TRα1, which is a classic ligand-binding receptor and TRα2 which is not capable of binding triiodothyronine (T_3_) and whose function is not yet clear (2). TRα1 is the predominant isoform in cardiac and skeletal muscle, the central nervous system, bone and inflammatory cells (3, 4, 5, 6, 7, 8, 9). There are two ligand-binding TRβ isoforms: TRβ1, which is mainly present in the brain, liver and kidney, and TRβ2, which is expressed in the hypothalamus and pituitary (1, 2).

Patients with resistance to TH due to mutations in TRβ (RTHβ) were first characterized decades ago. The first patients with inactivating mutations of the TH receptor α (TRα) were only recently identified (10, 11). Since then, 14 different mutations in the *THRA* gene that result in RTHα have been described to date (10, 11, 12, 13, 14, 15, 16, 17, 18, 19). Despite normal to only slightly abnormal plasma TH levels, clinical symptoms in these patients indicate resistance to TH at the tissue level including growth retardation, delayed bone development, constipation and cognitive defects (20). The severity of this phenotype is variable due to the heterogeneity of the underlying *THRA* mutations and their varying resultant loss of receptor function (12). The incidence of RTHα is expected to be similar to that of RTHβ, which is estimated to be around 1:40,000, due to the high degree of homology between the receptors (21).

Neutrophils and macrophages are both important phagocytic cells of the innate immune system. Neutrophils are the most abundant circulating leukocytes and, as the first cells to migrate to the site of infection, play an essential role in bacterial killing (22, 23). Macrophages are essential for the recruitment of other immune cells and can shape the immune response by eliciting either a pro-inflammatory or an anti-inflammatory reaction (24). Both neutrophils and macrophages are known to express TRα1 and other molecular elements of TH metabolism, including deiodinase enzymes (5, 6). Furthermore, intracellular TH metabolism has been linked to the immune function of these cells (25). Mice that lack TRα have higher levels of circulating pro-inflammatory cytokines at baseline (26), excessive secretion of pro-inflammatory cytokines by unstimulated macrophages (26, 27), a lower induction of the pro-inflammatory cytokine granulocyte-macrophage colony-stimulating factor (GM-CSF) during acute inflammation (5) and impaired macrophage function in an atherosclerosis model (26). These studies indicate that intracellular TH levels appear to play an important role in the function of innate immune cells. The mechanism behind these effects is currently unknown and is possibly mediated via the predominant TR in these cells: TRα.

Although THs do appear to affect immune function at the cellular level (25), the effect of hypothyroidism on the immune response in patients is not entirely clear. A number of studies suggest that hypothyroidism impairs the innate immune response (28, 29, 30); however, others have also described an increase in circulating pro-inflammatory cytokines in hypothyroid patients (31). Furthermore, T_3_ and T_4_ concentrations were positively correlated with several markers of inflammation in healthy euthyroid patients (32). The precise effects of altered thyroid status on the immune response *in vivo* remain to be determined.

The aim of this study is to determine whether TRα plays a role in the human innate immune response. To answer this, we assessed circulating pro-inflammatory cytokine levels in a previously described patient with RTHα (19) and found elevated concentrations of interleukin-8 (IL-8). We then measured IL-8 in a larger cohort of 8 RTHα patients, all of whom have been previously described (18, 19). To further study the role of TRα in specific innate immune cells, we measured neutrophil and macrophage function in a single RTHα patient and compared these results to healthy controls. This patient was identified after his daughter was found to be a carrier of a novel TRα mutation as described in a recent paper by van Gucht and coworkers (19). The patient was the only RTHα patient in our cohort who was not being treated with l-thyroxine at the time of study, which is important as the resistance of TRα to T_3_ can be overcome by high doses of T_3_ in the case of this mutation (19). The RTHα patients described here present a unique opportunity to determine whether a lack of TRα affects innate immune function in humans.

## Materials and methods

### Patients and controls

Sera from 8 previously described RTHα patients (18, 19) were obtained following written informed consent. These patients included two patients (one adult and one pediatric patient) with RTHαD211G as previously described by van Gucht and coworkers (19), 4 patients with RTHαA263S and 2 patients with RTHαR384H as previously described by Demir and coworkers, and van Gucht and coworkers (18, 19). With the exception of the adult RTHαD211G patient, all RTHα patients were undergoing treatment with l-thyroxine at the time of study. Neutrophil and macrophage function were assessed in cells derived from the adult RTHαD211G patient. This male patient (aged 31 years at the time of investigation) was identified as a carrier of a missense mutation D211G in TRα1 and TRα2 following the diagnosis of his daughter with the same mutation (19). The patient’s phenotype at diagnosis was reported previously (19). Briefly, the patient reported mild symptoms of tissue hypothyroidism including delayed puberty and constipation. Physical examination revealed coarse facies, macrocephaly, short stature and increased BMI. Blood pressure, bone mineral density and neuropsychological function tests were normal. Thyroid function tests at baseline are listed in Table 1. At the initiation of this study, the patient had never been treated with l-thyroxine. The patient reported no history of frequent bacterial infections or other signs of impaired innate immune function. Venous blood was also obtained from 11 healthy volunteers (6 males and 5 females, median age 29 years) following written informed consent. The study was approved by the Medical Ethical Committee of the Academic Medical Center Amsterdam in accordance with the principles of the Declaration of Helsinki (version Fortaleza, 2013).

**Table 1:** Thyroid hormone parameters of the RTHα patient. Values outside the reference range are indicated in bold.

**Variable**	**Reference values**	**Patient’s values**
T_4_	70–150 nmol/L	85
fT_4_	10–23 pmol/L	10.1
T_3_	1.3–2.7 nmol/L	2.25
rT_3_	0.11–0.44 nmol/L	0.12
TSH	0.5–5.0 mE/L	1.60
T_3_/T_4_ (×100)	1.42–3.05	2.65
T_3_/rT_3_	3.1–13.0	**18.75**
Thyroglobulin	0–45 pmol/L	13
IGF-1	8–41 nmol/L	24
Hemoglobin	8.5–10.5 mmol/L	**7.3**
MCV	80–100 fL	98.2
Ferritin	25–300 μg/L	272

Reproduced, with permission, from van Gucht AL, Meima ME, Zwaveling-Soonawala N, Visser WE, Fliers E, Wennink JM, Henny C, Visser TJ, Peeters RP & van Trotsenburg AS. Resistance to thyroid hormone alpha in an 18-month-old girl: clinical, therapeutic, and molecular characteristics, *Thyroid*, 2016, vol **26**, pages 338–346 (19).

### Cytokine measurements

Circulating levels of IL-8 were measured in sera from all RTHα patients and controls using the Human IL-8 Quantikine ELISA kit (R&D Systems) according to manufacturer’s instructions with the following modifications: sample volume was 100 µL, incubation time was 3 h and the following points were added to the standard curve: 3.6, 7.7, 15.8 and 31.3 pg/mL. Samples were measured in duplicate and samples below the detection limit (7.7 pg/mL) were assigned a value of half the detection limit.

A panel of pro-inflammatory cytokines and chemokines (IL-1β, IL-6, TNF and IL-8) were measured in supernatant of stimulated macrophages and in plasma from the adult RTHαD211G patient and healthy controls using the Human Inflammatory Cytokine Cytometric Bead Array kit (BD Biosciences, Franklin Lakes, NJ, USA). Samples were run in triplicate on a FACS Calibur flow cytometer (BD Biosciences). All samples were measured in the same run. Data were analyzed using FlowJo software (version 10).

### Cell isolation and culture

Neutrophils were isolated as described previously (33, 34). Briefly, heparinized venous blood was subjected to density gradient centrifugation over isotonic Percoll (1.076 g/mL). Peripheral blood mononuclear cells (PBMC’s) were collected and used for monocyte isolation (see below). The pellet containing erythrocytes and granulocytes was harvested. Following erythrocyte lysis, neutrophils were washed and re-suspended in HEPES-buffered medium (132 mM NaCl, 6 mM KCl, 1 mM CaCl_2_, 1 mM MgSO_4_, 1.2 mM K_2_HPO_4_, 20 mM HEPES, 1 mg/mL glucose, and 0.5% (wt/vol) human serum albumin, pH 7.4). Cells were kept at room temperature (RT) until use. Neutrophil purity was assessed using flow cytometry.

Monocytes were separated from PBMCs by positive selection using a MACS magnetic cell separation kit in combination with anti-CD14 magnetic beads (Miltenyi Biotec, Leiden, the Netherlands) according to manufacturer’s instructions. Monocytes were washed and re-suspended in differentiation medium (RPMI-1640 medium (Lonza, Basel, Switzerland) with 2% human pooled AB serum (Sigma Aldrich), 2.5 ng/mL of human M-CSF (eBioscience, San Diego, CA, USA) and 10 U/mL of penicillin and streptomycin (Lonza) plated at 1 × 10^6^/mL) (35). Cells were cultured at 37°C and 5% CO_2_ for 7 days. On day 7, differentiation medium was removed and cells were subsequently cultured in RPMI-1640 medium (Lonza) with 10% fetal calf serum. Macrophage differentiation was assessed visually. Macrophage purity was checked using flow cytometry.

### Neutrophil bacterial killing

Neutrophil *in vitro* bacterial killing of *Escherichia coli* (strain ML-35) and *Staphylococcus aureus* (strain 502A) was measured as described previously (36, 37). Briefly, bacteria were grown aerobically at 37°C until logarithmic growth was reached. Bacteria were washed and re-suspended at an OD 600 of 1 (i.e. 109 bacteria/mL). After opsonization, bacteria were added to neutrophils at a ratio of 5:1 and incubated at 37°C for the indicated time period. At the desired time points, samples were taken and neutrophils were lysed in water (pH 11.0). Serial dilutions of lysates were plated and incubated at 37°C overnight after which colony-forming units (CFU) were counted from which the percentage of neutrophil bacterial killing was calculated. The bacterial killing assay with neutrophils from the TRα-deficient patient were run in parallel with a day control and compared to a preexisting database of healthy controls (*n* = 32 for *E. coli* and *n* = 36 for *S. aureus*).

### Neutrophil NADPH oxidase activity and chemotaxis

Nicotinamide adenine dinucleotide phosphate-oxidase (NADPH oxidase) activity was measured as described previously (38). Briefly, extracellular hydrogen peroxidase (H_2_O_2_) release in response to stimuli was measured using the Amplex Red (10-acetyl-3,7-dihydroxyphenoxazine) Hydrogen Peroxidase Assay kit (Molecular Probes). Phorbol 12-myristate 13-acetate (PMA, 100 ng/mL), unopsonized zymosan (1 mg/mL), serum-treated zymosan (STZ, 1 mg/mL), platelet-activating factor (PAF, 1 μM) followed by formyl-Met-Leu-Phe (fMLP, 1 μM) were used as stimuli (all Sigma Aldrich). Fluorescence was measured at 30-s intervals for 20 min with the Infinite 200 PRO (Tecan, Mannedorf, Switzerland). Results were compared to a day control and to the normal range of historical controls (*n* = 162).

Neutrophil migration toward various chemotactic stimuli was measured using 3 µm pore-size Fluoroblock inserts (Corning), in a Falcon 24-well plate as described previously (39). Neutrophils were fluorescently labeled with calcein AM (Thermo Fisher Scientific) and the following stimuli were used: complement component 5a (C5a), interleukin 8 (IL-8) and PAF. Results were compared to a day control, and to the normal range of historical controls (*n* = 132).

### Neutrophil apoptosis

Spontaneous apoptosis was assessed in freshly isolated unstimulated neutrophils. Cells were incubated in a shaking water bath at 37°C for up to 24 h. Samples were harvested at the appropriate time points and double stained for Annexin V and propidium iodide (both BD Biosciences) according to manufacturer’s instructions. Samples were acquired on a BD FACS Canto II flow cytometer, and data were analyzed using FlowJo software (v.10).

### Macrophage phagocytosis

Differentiated macrophages were incubated in a 96-well plate (5 × 10^4^/well) with opsonized pHrodo green zymosan BioParticles conjugate (Molecular Probes) for 2 h at 37°C. pHrodo is a fluorogenic dye that strongly increases in fluorescence as the pH of its surroundings decreases. Since the extracellular environment is at a neutral pH and the intraphagosomal environment is highly acidic, the amount of fluorescence generated is an indirect measure for the amount of phagocytosed particles. Fluorescence was quantified on a Varioskan Flash plate reader (Thermo Fisher Scientific).

### Macrophage stimulation, RNA isolation and qPCR

Differentiated macrophages were incubated with or without 100 ng/mL lipopolysaccharide (LPS or bacterial endotoxin, *Escherichia coli* strain 055:B5; Sigma Aldrich) for 3 h after which medium was harvested for cytokine measurements (see below) and cells were processed for RNA isolation using the High Pure RNA isolation kit (Roche). cDNA was synthesized with equal RNA input using AMV Reverse Transcriptase enzyme with oligo d(T) primers (Roche). A cDNA synthesis reaction without reverse transcriptase was included as a control for genomic DNA contamination. Quantitative real-time PCR was carried out using the Lightcycler 480 (Roche) and SensiFAST SYBR No-ROX (Bioline, Taunton, MA, USA). Data were analyzed using LinReg software. The mean of the efficiency was calculated for each assay, and samples that deviated more than 0.05 of the efficiency mean value were excluded from the analysis (0–5%). Primer sequences for *HPRT1* (hypoxanthine phosphoribosyltransferase 1 (HPRT)), *TNF* (tumor necrosis factor α (TNFα)) and *CXCL8* (interleukin-8 (IL-8)) were published previously (40, 41, 42, 43). Primer sequences for *IL1B* (interleukin-1β (IL-1β)) and *IL6* (interleukin-6 (IL-6)) were derived from the Harvard Primer Bank (numbers 221139821c1, 27894305c1 and 224831235c1, respectively). Primers were newly designed for the reference gene *EEF1A1* (eukaryotic translation elongation factor 1 alpha 1; Ef1α1: forward primer 5′-TTTTCGCAACGGGTTTGCC-3′, reverse primer: 5′-TTGCCCGAATCTACGTGTCC-3′, annealing temperature 65°C). Calculated values were normalized using the geometric mean of the reference genes Ef1α1 and HPRT.

### Statistics

Statistical analysis was performed in GraphPad Prism, version 7.01. Differences in serum IL-8 levels between controls and RTHα patients were tested using an unpaired Student’s *t*-test. *P* < 0.05 was considered significant. Due to the study design, it was not possible to test whether differences in neutrophil and macrophage function between the single RTHα patient, and the healthy controls were statistically significant. Instead, we determined whether the results from the RTHα patient were within the range of normal controls. If values were outside this range, control data were tested for normality using the Shapiro–Wilk test. In the case of normal distribution, a *z*-score was calculated for the RTHα values. *Z*-scores that were >1.96 or <−1.96 (significance levels for *α* = 0.05) are listed.

## Results

### RTHα leads to an increase in circulating IL-8 concentrations

Plasma levels of selected pro-inflammatory cytokines and chemokines were measured in samples from both healthy controls and the adult RTHαD211G patient. IL-1β, IL-6, TNF, IL12p70 and IL-10 were below the detection limit of our assay (2.5–5 pg/mL) in all samples. However, we were able to detect IL-8 in both plasma of healthy controls and plasma from the RTHα patient. Plasma IL-8 in the RTHα patient was found to be higher than that in the healthy control group.

**Figure 1:**
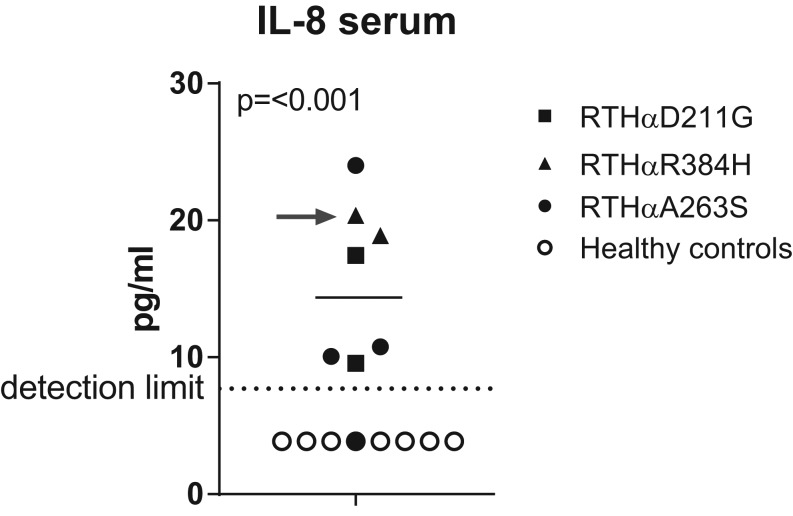
Serum IL-8 levels are increased in RTHα. IL-8 concentrations were quantified using an ELISA in serum samples from RTHα patients (filled symbols, *n* = 8) and healthy controls (○, *n* = 8). CRP levels were measured in the same serum sample. The detection limit of the assay is indicated (7.7 pg/mL). Samples below this limit were assigned a value of half of the detection limit (3.85 pg/mL). CRP levels were within the normal range (<5 mg/L) in all samples with the exception of one RTHαR384H patient (indicated with gray arrow) who had a slightly elevated CRP of 7.8 mg/L without clinical signs of illness. The mean of the RTHα samples is indicated. The *P* value indicated represents an unpaired Student’s *t*-test.

Following this finding, we measured IL-8 in sera from a cohort of previously described RTHα patients with different causative mutations to determine whether increased circulating IL-8 was a general feature of RTHα or specific to the D211G mutation. In total, sera from 7 additional patients were obtained. These patients included another patient with RTHαD211G as previously described by van Gucht and coworkers (19), 4 patients with RTHαA263S and 2 patients with RTHαR384H all as previously described by Demir and coworkers, and van Gucht and coworkers (18, 19). IL-8 was measured in all of the additional samples, together with samples from the original patient and controls. IL-8 was found to be below the detection limit in all healthy controls and significantly elevated in RTHα patients (Fig. 1). Both healthy controls and RTHα patients reported no signs of illness on the day of blood draw. C-reactive protein (CRP), a highly specific determinant of inflammation, was measured in the same serum sample in which IL-8 was measured. One RTHα patient had a slightly elevated CRP of 7.8 mg/L (Fig. 1, reference value: <5 mg/L). All the remaining controls and patients had CRP concentrations within the normal range.

### Neutrophil survival, bacterial killing, H_2_O_2_ production and chemotaxis are unaffected in RTHα

To determine whether RTHα affected not only circulating cytokine levels, but also innate immune cell function, we measured neutrophil and macrophage function in the adult RTHαD211G patient and healthy controls.

**Figure 2:**
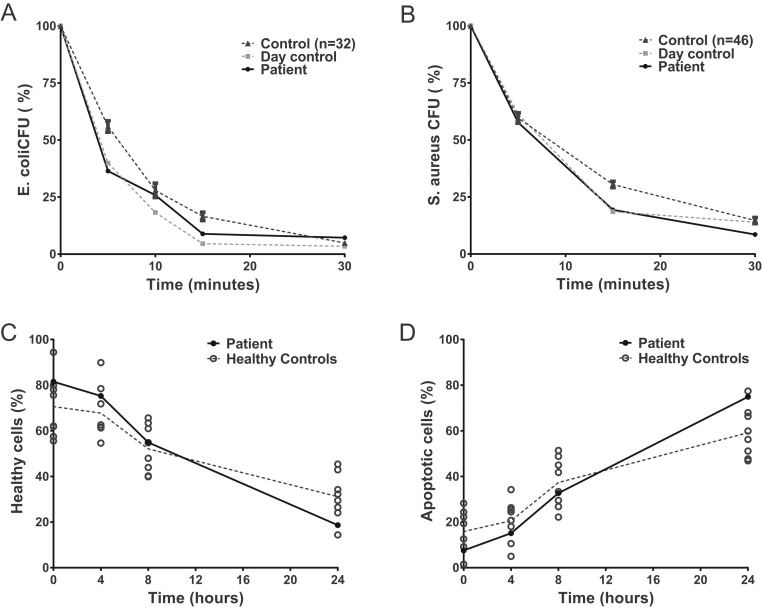
RTHα neutrophils show unchanged bacterial killing and survival *ex vivo*. (A) and (B) Freshly isolated neutrophils were incubated with live opsonized *E. coli* (A) or *S. aureus* (B) at 37°C. Graphs indicate the remaining percentage of bacteria present at the indicated time points vs baseline levels. RTHα neutrophils were run in parallel with a day control. Previously acquired controls values are also shown. (C) and (D) Freshly isolated neutrophils were incubated at 37°C. Samples were taken at the indicated time points and double stained for Annexin V and propidium iodide, markers for apoptosis and cell death, respectively. The percentage of healthy cells (C) and the percentage of Annexin V-positive, or apoptotic, cells (D) are indicated over time.

**Figure 3:**
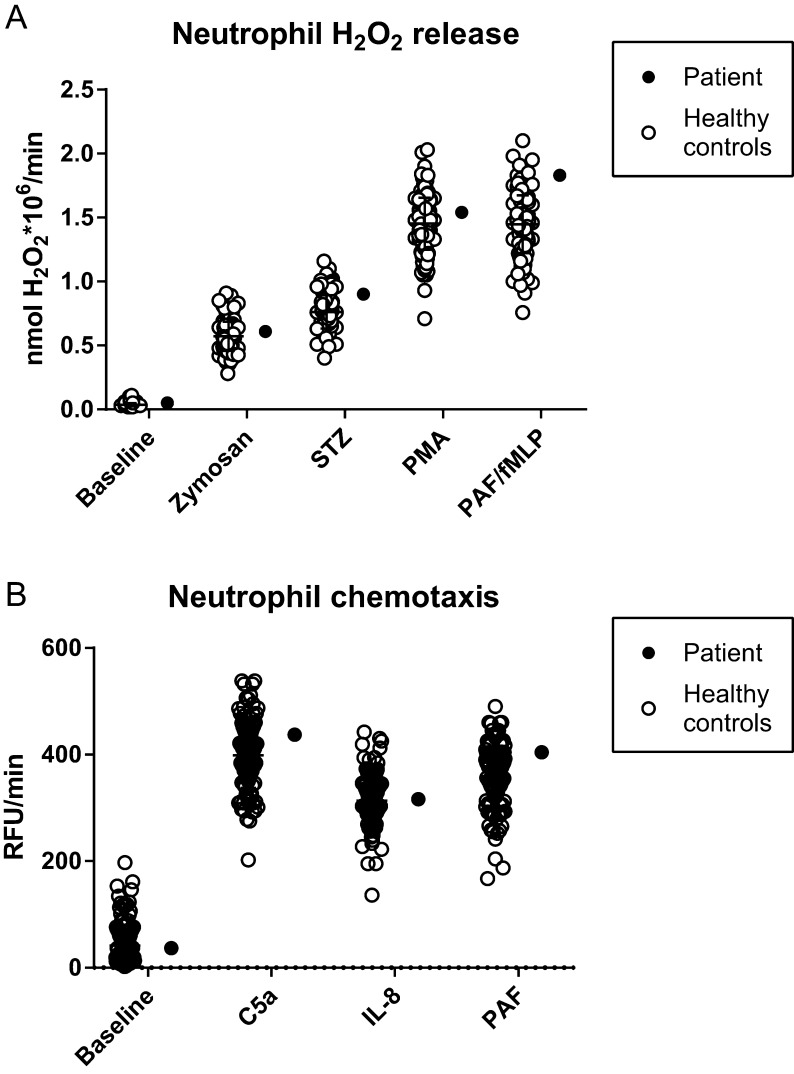
Neutrophil H_2_O_2_ release and chemotaxis are unchanged in an RTHα patient. (A) Neutrophil hydrogen peroxide (H_2_O_2_) release in response to stimuli. STZ, serum-treated zymosan; PMA, phorbol 12-myristate 13-acetate; PAF, platelet-activating factor; fMLP, formyl-Met-Leu-Phe. Mean ± s.d. is indicated for data from healthy controls. (B) Migration of fluorescently labeled neutrophils toward various chemotactic stimuli. C5a, complement component 5a; IL-8, interleukin 8; PAF, platelet-activating factor. Data are indicated in relative fluorescent units (RFU) per minute. Mean ± s.d. is indicated for data from healthy controls.

Various important neutrophil effector functions were analyzed in neutrophils derived from the RTHαD211G patient and cells derived from healthy controls. Neutrophils with an inactivating TRα mutation were incubated with live *E. coli* and *S. aureus* and showed normal bacterial killing compared to a previously acquired dataset of controls and a day control run in parallel (Fig. 2A and B). Spontaneous neutrophil apoptosis (i.e. neutrophil lifespan) was also unchanged in RTHα neutrophils when compared to neutrophils derived from controls (Fig. 2C and D). The ability of RTHα neutrophils to migrate toward the chemotactic stimuli C5a, IL-8 and PAF was within the normal range, as was their ability to produce H_2_O_2_ upon stimulation with various pro-inflammatory stimuli (Fig. 3). H_2_O_2_ production is a measure for NADPH oxidase activity, which is an essential component of the neutrophil bacterial killing machinery (23). In conclusion, RTHα in this patient does not result in changes in the ability of neutrophil to migrate toward, recognize, phagocytose and kill bacteria.

### Pro-inflammatory macrophage function is not altered in RTHα

**Figure 4:**
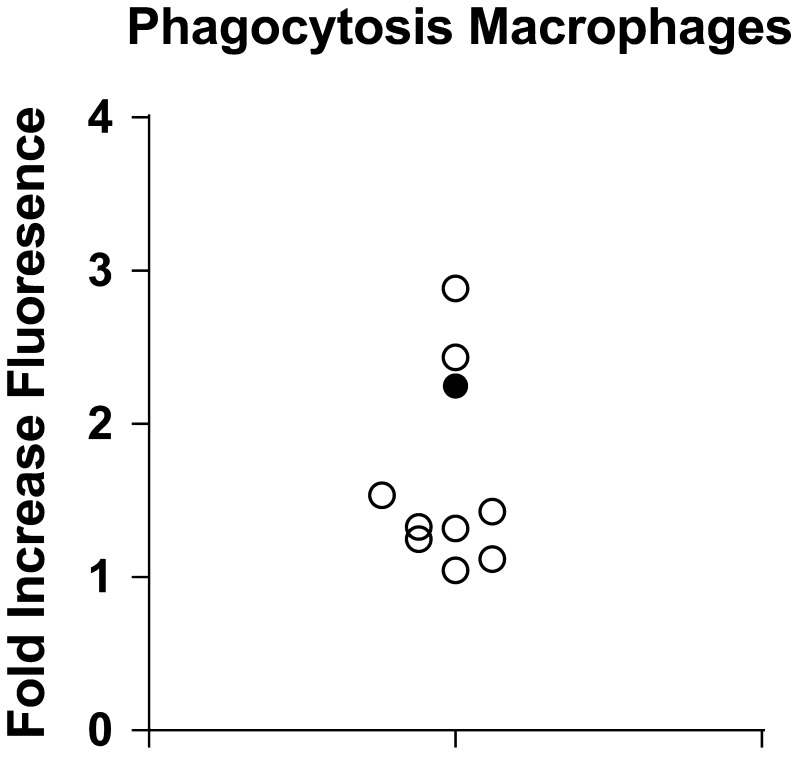
Macrophage phagocytosis is unchanged in an RTHα patient. Macrophages from the RTHα patient (●) and healthy controls (○) were incubated with pHrodo-labeled zymosan (yeast particles) for 2 h at 37°C. pHrodo becomes fluorescent at a low pH such as that present in phagosomes. The fold increase in relative fluorescent units vs pHrodo-labeled zymosan alone is shown.

**Figure 5:**
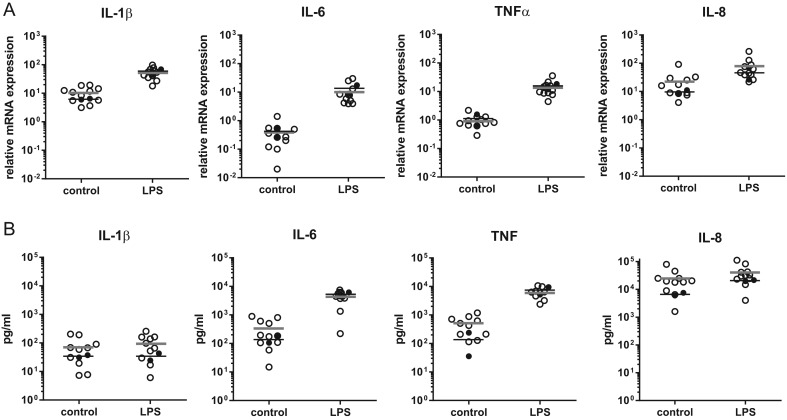
Macrophage pro-inflammatory cytokine levels are unchanged at baseline and after LPS stimulation in an RTHα patient; Macrophages from the RTHα patient (●) and healthy controls (○) were incubated with or without LPS (100 ng/mL) for 3 h. Cytokine relative mRNA expression (A) and secreted protein concentrations (B) are depicted. For the RTHα patient results from two independent experiments are shown.

Several essential aspects of pro-inflammatory macrophage function were measured in macrophages derived from the RTHαD211G patient and healthy controls. Phagocytosis, determined by the cells ability to engulf fluorescent particles, was found to be unchanged in RTHα macrophages compared to control macrophages (Fig. 4). Macrophages were also stimulated with LPS, a bacterial cell wall component that acts as a strong pro-inflammatory stimulus. LPS stimulation resulted in a robust induction of the pro-inflammatory cytokines IL-1β, IL-6, TNFα and IL-8 at the transcriptional level (Fig. 5A) and at the protein level (Fig. 5B). The response in RTHα macrophages was within the range of healthy control cells, both at the transcriptional and at the secretory/protein level (Fig. 5).

## Discussion

Innate immune cells have recently been identified as novel TH target cells (25). It is currently unknown whether the effects of TH in innate immune cells are mediated via TRα, the predominant TR isoform in these cells. The aim of this study is to determine whether TRα plays a role in innate immune function in humans. More insight into the effects of an inactivating TRα mutation can lead to improved future treatment of RTHα patients, and greatly increase the understanding of this syndrome and its clinical and physiological consequences. We are the first to study the effects of RTHα on innate immunity in humans.

We find elevated levels of circulating IL-8 in RTHα patients. IL-8, also known as CXCL8, is a potent pro-inflammatory chemokine whose primary function is to recruit and activate inflammatory cells, mainly neutrophils, to the site of infection via a chemotactic gradient (44). IL-8 is expressed in humans, but there is no rodent equivalent (44). As IL-8 expression and secretion were not elevated in RTHα macrophages, the increase in circulating IL-8 is most likely due to increased production by another cell type. Interestingly, elevated circulating IL-8 levels have also been described in hyperthyroidism (both Graves’ disease and toxic multinodular goiter) (45). Furthermore, T_3_ induces IL-8 production in bone marrow stromal cells and a human osteoblast cell line (46), suggesting that the increase in circulating IL-8 could be a consequence of high circulating T_3_ concentrations, rather than a cause of autoimmune thyroid disease. RTHα patients tend to have high-normal to high levels of circulating T_3_ (12); this could potentially result in increased levels of IL-8. As elevated IL-8 was observed in patients both on and off levothyroxine, it appears to be an effect of the underlying condition, not its treatment.

Intracellular TH metabolism is thought to play an important role in the bacterial killing abilities of neutrophils via the induction of type 3 deiodinase (D3) (6, 25, 47, 48, 49). The mechanism behind this remains unclear (25). One of the possibilities is that the modulation of intracellular T_3_ levels by D3 could result in an effect through changes in TR occupancy and subsequent T_3_-dependent gene transcription. TRα is the predominant receptor isoform in both neutrophils and macrophages (5, 6). In macrophages, intracellular T_3_ availability and action also appear to be important for pro-inflammatory function (5, 25). Our results in this patient suggest that the effects of intracellular TH metabolism on neutrophil and macrophage function are not mediated via the TRα. However, as the D211G mutation is a relatively mild mutation, in which TRα1 has reduced transcriptional activity which can be overcome by high concentrations of T_3_ (100 nM (19)), we cannot exclude the possibility that some transcriptional activity of the receptor is preserved in cells from this RTHα patient. Another possible explanation could be that the effects of T_3_ in these cells are mediated via pathways that do not require binding to the TR, such as the signaling pathway involving the plasma membrane integrin receptor avβ3 (50). Other authors have suggested that the effects of extracellular TH on macrophages are mediated via this receptor, resulting in the activation of the ERK1/2 and PI3K pathways (51). Whether these pathways could also be involved in intracellular TH signaling is currently unknown.

Interestingly, macrophages derived from TRα^0/0^ mice do exhibit altered function. TRα^0/0^ mice show deficient macrophage cholesterol efflux, increased aortic inflammation, elevated serum pro-inflammatory cytokine levels and increased macrophage pro-inflammatory cytokine expression and secretion (26, 27). However, we do not find changes in macrophage cytokine induction in human macrophages derived from an RTHα patient compared to healthy controls. This discrepancy could be due to the fact that TRα^0/0^ mice are completely deficient for TRα, whereas RTHα patients exhibit decreased sensitivity for T_3_ but retain the dominant negative activity of the receptor (52, 53).

The main limitation of the functional neutrophil and macrophage assays in this study is the fact that material from only one untreated RTHα patient was studied. The functional leukocyte assays using RTHα leukocytes were repeated independently yielding similar results; we therefore believe that the lack of phenotypical abnormalities in RTHα neutrophils and macrophages is consistent, at least in the case of the D211G mutation. However, as mutations resulting in RTHα are heterozygous, we cannot exclude that other TRα mutations, with for example, a more severe loss of receptor function, might affect leukocyte function. As leukocytes need to be isolated from heparinized venous blood within several hours after the blood draw, obtaining and analyzing cells from larger numbers of patients is logistically very complicated due to the very small number of currently available untreated RTHα patients. However, as we were able demonstrate elevated IL-8 in serum samples from a number of other RTHα patients harboring different mutations, we believe this considerably strengthens the study and confirms that increased IL-8 is a general feature of RTHα and not one limited to the D211G mutation.

Intracellular TH metabolism has been shown to be essential for adequate pro-inflammatory neutrophil and macrophage function, identifying innate immune cells as novel TH target cells (25). This study is the first to assess the role of TRα in human innate immune function. Our results show that RTHα results in an increase in circulating IL-8 levels, which has also been described in hyperthyroid patients and therefore might lead to a slight increase in circulating T_3_. Furthermore, we demonstrate that a relatively mild mutation in the TRα does not result in clinically relevant impairment of neutrophil or pro-inflammatory macrophage function.

## Declaration of interest

The authors declare that there is no conflict of interest that could be perceived as prejudicing the impartiality of the research reported.

## Funding

This work was supported by the AMC Graduate School PhD Scholarship (A H v d S).

## Author contribution statement

A H v d S, E F and A B designed the experiments. A H v d S, O V S, S A, A T J T, A v d G and A L M v G performed the experiments. K D and A S P v T provided patient material. T K v d B, E F and A B supervised experiments. A H v d S, E F and A B wrote the original draft of the manuscript. All authors read and approved the final version of the manuscript.
